# Optical and Structural Properties of Composites Based on Poly(urethane) and TiO_2_ Nanowires

**DOI:** 10.3390/ma16041742

**Published:** 2023-02-20

**Authors:** Malvina Stroe, Teodora Burlanescu, Mirela Paraschiv, Adam Lőrinczi, Elena Matei, Romeo Ciobanu, Mihaela Baibarac

**Affiliations:** 1 National Institute of Materials Physics, P.O. Box MG-7, Bucharest, Atomistilor Street 405A, 077125 Bucharest, Romania; 2 SC All Green SRL, 8 George Cosbuc Str., 700470 Iasi, Romania; 3 Electrical Engineering Faculty, Gheorghe Asachi Technical University of Iasi, Dimitrie Mangeron Bd. 67, 700050 Iasi, Romania

**Keywords:** TiO_2_ nanowires, poly(urethane), composites

## Abstract

This article’s objective is the synthesis of new composites based on thermoplastic polyurethane (TPU) and TiO_2_ nanowires (NWs) as free-standing films, highlighting their structural and optical properties. The free-standing TPU–TiO_2_ NW films were prepared by a wet chemical method accompanied by a thermal treatment at 100 °C for 1 h, followed by air-drying for 2 h. X-ray diffraction (XRD) studies indicated that the starting commercial TiO_2_ NW sample contains TiO_2_ tetragonal anatase (A), cubic Ti_0.91_O (C), and orthorhombic Ti_2_O_3_ (OR), as well as monoclinic H_2_Ti_3_O_7_ (M). In the presence of TPU, an increase in the ratio between the intensities of the diffraction peaks at 43.4° and 48° belonging to the C and A phases of titanium dioxide, respectively, is reported. The increase in the intensity of the peak at 43.4° is explained to be a consequence of the interaction of TiO_2_ NWs with PTU, which occurs when the formation of suboxides takes place. The variation in the ratio of the absorbance of the IR bands peaked at 765–771 cm^−1^ and 3304–3315 cm^−1^ from 4.68 to 4.21 and 3.83 for TPU and the TPU–TiO_2_ NW composites, respectively, with TiO_2_ NW concentration equal to 2 wt.% and 17 wt.%, indicated a decrease in the higher-order aggregates of TPU with a simultaneous increase in the hydrogen bonds established between the amide groups of TPU and the oxygen atoms of TiO_2_ NWs. The decrease in the ratio of the intensity of the Raman lines peaked at 658 cm^−1^ and 635 cm^−1^, which were assigned to the vibrational modes E_g_ in TiO_2_ A and E_g_ in H_2_Ti_3_O_7_ (I_TiO2-A_/I_H2Ti3O7_), respectively, from 3.45 in TiO_2_ NWs to 0.94–0.96 in the TPU–TiO_2_ NW composites, which indicates that the adsorption of TPU onto TiO_2_ NWs involves an exchange reaction of TPU in the presence of TiO_2_ NWs, followed by the formation of new hydrogen bonds between the -NH- of the amide group and the oxygen atoms of Ti_x_O_2x-mn_, Ti_2_O_3_, and Ti_0.91_O. Photoluminescence (PL) studies highlighted a gradual decrease in the intensity of the TPU emission band, which is situated in the spectral range 380–650 nm, in the presence of TiO_2_ NW. After increasing the TiO_2_ NW concentration in the TPU–TiO_2_ NW composite mass from 0 wt.% to 2 wt.% and 17 wt.%, respectively, a change in the binding angle of the TPU onto the TiO_2_ NW surface from 12.6° to 32° and 45.9°, respectively, took place.

## 1. Introduction

Various conformations of TiO_2_ particles, such as nanowires, nanorods, and nanobelts, have been synthesized [1]. The protocols used for the synthesis of TiO_2_ particles were: (i) hydrothermal/solvothermal [2]; (ii) sol–gel synthesis [3]; (iii) surfactant-assisted [4]; (iv) microwave-assisted [5]; (v) sonochemical synthesis [6]; (vi) high-temperature pyrolysis [7]; (vii) electrospinning [8]; (viii) chemical/physical vapor deposition [9,10]; (ix) atomic layer deposition [11]; (x) pulsed laser deposition [12]; (xi) suspended molecular template [13]; and (xii) electrochemical deposition [1,14]. That used by Sigma-Aldrich for the synthesis of TiO_2_ NWs was the hydrothermal method. Other conformations of TiO_2_ particles, such as nanocubes [5], nanospheres [5], nanorods [5], nanowires [15], nanotubes [16], nanosheets [17], nanobelts [18], etc., were reported. Considerable information concerning the polycrystallinity of TiO_2_ was discovered by X-ray diffraction in 2016 [19]. The main crystalline phases reported in the case of TiO_2_ nanoparticles were rutile [20], anatase [21], and brookite [22]. The band gap of TiO_2_ nanoparticles with a rutile-, anatase-, or brookite-type crystalline phase was of 1.78 eV, 2.04 eV, and 2.2 eV, respectively [23]. One of the most considerable applications of TiO_2_ was in the field of photocatalysis [24]. To enhance the photocatalytic properties of TiO_2_, different strategies have been adopted for doping TiO_2_ with metals and non-metals, as recently reported by P.S. Basavarajappa et al. [24]. To develop other applications, special attention was paid to the synthesis of composites based on TiO_2_ particles and polymers of types such as poly(vinylidene fluoride) [25], polyacrylonitrile [26], polypyrrole–chitosan [27], fluor-polydopamine [28], poly(3-methyl thiophene) [29], poly(3-hexyl thiophene) [30], poly(3,4-ethylenedioxythiopene):poly(2-styrene sulfonate) [31], polyaniline [32], and thermoplastic polyurethane [33]. The main applications of composites based on thermoplastic polyurethane (TPU) and TiO_2_ nanoparticles were reported in Li-ion batteries [34], binder for asphalt [35], water filters [36], the adsorption of oils spilled in water [37], and photocatalysis [38]. For such applications, composites containing TiO_2_ nanoparticles with anatase- (A) [33,35,39] and rutile-type (R) [40] crystalline phases were used. The synthesis methods used to prepare the TPU–TiO_2_ composites included phase inversion [34], mixing in the melting [39], and the wet-spinning process [38]. The most used methods for the characterization of the TPU–TiO_2_ composites were X-ray diffraction (XRD) [34], FTIR spectroscopy [34,35,39,40], scanning electron microscopy (SEM) [37,40], and thermogravimetry [38,39]. Cross-linked composites reportedly resulted from the interaction between TPU with TiO_2_ nanoparticles and a type-R crystalline phase [40]. Compared with the progress made in the literature, our work was focused on the synthesis of composites based on TPU and TiO_2_ nanowires (NWs) and then on highlighting their structural and optical properties by XRD, Raman scattering, FTIR spectroscopy, photoluminescence, scanning electron microscopy (SEM), and energy-dispersive X-ray spectroscopy (EDS). Our motivation for this study was to consider the possibility of using a TPU–TiO_2_ NW composite in the field of ventricular catheters, which are currently manufactured from poly(dimethyl)siloxane (PDMS) and polyurethanes (PU) [41,42]. The main disadvantages of these ventricular catheters are protein adsorption, shunt obstruction, and the appearance of infections [41,42]. The interaction of polymers with compounds with increased hydrophilicity and photocatalytic properties, such as TiO_2_, can overcome these inconveniences. Consequently, our aim was the synthesis of TPU–TiO_2_ NW composites, highlighting the potential interactions between the two constituents of these composites. Our preliminary results will open new opportunities for optimizing these composites and evaluating their performance in the field of ventricular catheters.

Here, we used XRD to uncover information about the crystalline planes of TiO_2_ NWs. We show that the NWs contain tetragonal TiO_2_ anatase (A), cubic Ti_0.91_O (C), orthorhombic Ti_2_O_3_ (OR), and monoclinic H_2_Ti_3_O_7_ (M). To highlight the potential changes in the chemical structure of TPU and TiO_2_ NWs, we show the correlated studies by Raman scattering and FTIR spectroscopy. According to our previous study, these characterization methods are valuable tools to highlight the exchange reaction of TPU in the presence of BaTiO_3_ nanoparticles [43]. Using scanning electron microscopy (SEM), we show the fibrous structure of the TPU–TiO_2_ NW composites. To assess the binding angle of TPU onto the TiO_2_ NW surface, we performed anisotropic PL measurements. Our research allows an understanding of TPU’s adsorption process onto the TiO_2_ NW surface. Recently, we demonstrated that TPU shows, at an excitation wavelength of 350 nm, a photoluminescence (PL) band with maximum at 410 nm [43]. We also analyzed the influence of TiO_2_ NWs on TPU PL properties.

## 2. Materials and Methods

### 2.1. Materials

TPU was purchased from the Elastollan-BASF Chemical Company (Cleveland, OH, USA), while TiO_2_ NWs and N,N′-dimethyl formamide (DMF, 99.8%) were purchased from Sigma-Aldrich (St. Louis, MO, USA). According to the TiO_2_ NWs specification sheet, the diameter and length of the NWs were ~10 nm and ~10 μm.

### 2.2. Synthesis Method of TPU–TiO_2_ NW Composites

The free-standing-film TPU–TiO_2_ NW composites were prepared by the wet chemistry method as follows: (a) we dissolved 0.5 g TPU in 16 mL DMF under ultrasonication; (b) in each solution of TPU in DMF (0.5 g/16 mL), we added 50 or 100 mg of TiO_2_ NW; (c) the dispersion of TiO_2_ particles in the TPU solution was performed under ultrasonication for 20 min; (d) the TiO_2_ suspensions in the solutions of TPU in DMF were poured into a petri vessel and subjected to a thermal treatment for 1 h at a temperature of 100 °C for DMF evaporation; and (ed) we dried the TPU samples with different TiO_2_ concentrations, i.e., 2 wt.% and 17 wt.%, in air for 2 h until the free-standing films’ mass remained constant.

The TPU free-standing films were prepared as above without adding the TiO_2_ NWs.

**Figure 1:**
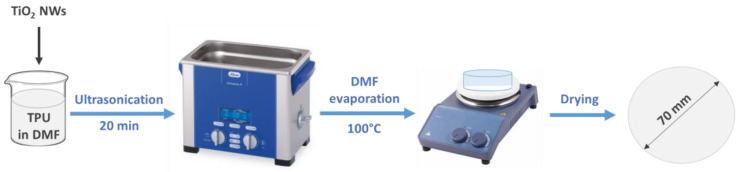
Synthesis method for TPU–TiO_2_ NW composites as free-standing films.

Figure 1 shows the schematic synthesis method for TPU–TiO_2_ NW composites as free-standing films.

### 2.3. Methods

#### 2.3.1. X-ray Diffraction Analysis

The XRD patterns of the TiO_2_ NWs and TPU–TiO_2_ NW composites, which have a TiO_2_ NW concentration equal to 2 wt.% and 17 wt.%, respectively, were carried out using a Bruker D8 Advance diffractometer (Bruker, Hamburg, Germany) in Bragg–Brentano geometry, which was equipped with a Cu tube, and which had a Cu K_α_-line of λ = 1.5406 Å.

#### 2.3.2. Fourier-Transform Infrared (FTIR) Spectroscopic Analysis

The IR spectra of TPU and the TPU–TiO_2_ NW composites, which had TiO_2_ NW concentrations equal to 2 wt.% and 17 wt.%, respectively, were recorded using an FTIR spectrophotometer Vertex 80 model from Bruker (Billerica, MA, USA).

#### 2.3.3. FT-Raman Spectroscopic Analysis

The Raman spectra of TPU and the TPU–TiO_2_ NW composites, which have TiO_2_ NW concentrations equal to 2 wt.% and 17 wt.%, respectively, were recorded with an FT-Raman spectrophotometer MultiRam model from Bruker at an excitation wavelength of 1064 nm (Ettlingen, Germany).

#### 2.3.4. Photoluminescence Analysis

The photoluminescence (PL) spectra of TPU and the TPU–TiO_2_ NW composites, which have TiO_2_ NW concentrations equal to 2 wt.% and 17 wt.%, respectively, were recorded with a Fluorolog-3 spectrophotometer FL3-2.2.1 model from Horiba Jobin Yvon (Palaiseau, France).

#### 2.3.5. Scanning Electron Microscopy and Energy-Dispersive X-Ray Analysis

Scanning electron microscopy (SEM) and energy-dispersive X-ray (EDS) analysis of TPU and the TPU–TiO_2_ NW composites, which have TiO_2_ NW concentrations equal to 2 wt.% and 17 wt.%, respectively, were achieved with a Zeiss Gemini 500 field-emission scanning electron microscope and a Zeiss EVO 50 XVP system (Zeiss, Oberkochen, Germany) equipped with a Bruker EDS detector, respectively.

## 3. Results and Discussion

### 3.1. Morphological Properties of TiO_2_ NWs and the TPU–TiO_2_ Composites

**Figure 2:**
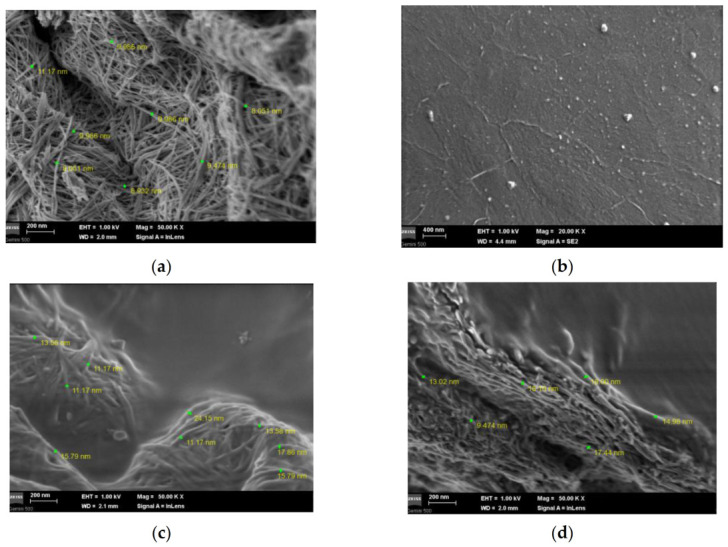
The SEM images of TiO_2_ NWs (**a**), as well as TPU (**b**) and the TPU–TiO_2_ NW composites, which have TiO_2_ NW concentrations equal to 2 wt.% (**c**) and 17 wt.% (**d**), respectively.

Figure 2 shows the SEM images of TiO_2_ NWs, as well as TPU and the TPU–TiO_2_ NW composites, which have TiO_2_ NW concentrations equal to 2 wt.% and 17 wt.%, respectively.

**Figure 3:**
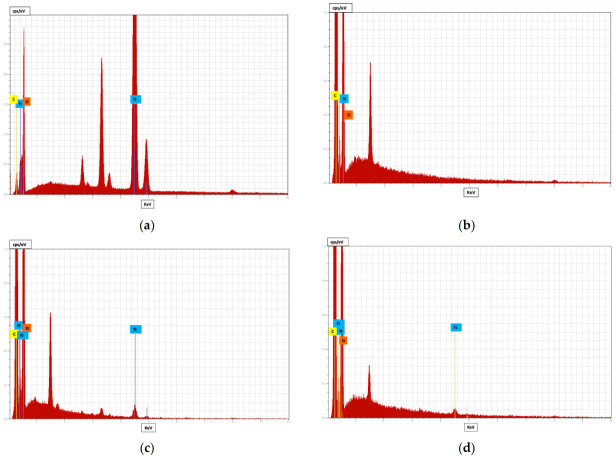
The EDS spectra of TiO_2_ NWs (**a**), TPU (**b**) and the TPU–TiO_2_ NW composites having the TiO_2_ NW concentration equal to 2 wt.% (**c**) and 17 wt.% (**d**). The unassigned maxima belong to the sample fixation substrate.

According to Figure 2a, the TiO_2_ NWs diameter varies between 8 and 11 nm. In contrast with Figure 2b, which shows an SEM image of TPU, in the case of Figure 2c,d, one can observe that the TPU–TiO_2_ NW composites show a fibrous structure, while the TiO_2_ NW diameter varies in the case of the TPU–TiO_2_ NW composites that have TiO_2_ NW concentrations of 2 wt.% and 17 wt.%, respectively, between 11 and 24 nm and 9 and 18 nm, respectively. The apparent increase in the diameter of TiO_2_ NWs is caused by the adsorption of polymer on the surface of TiO_2_ nanoparticles. Figure 3 shows the EDS spectra of TiO_2_ NWs, as well as the TPU and the TPU–TiO_2_ NW composites, which have a TiO_2_ NW concentration equal to 2 wt.% (c) and 17 wt.%, respectively.

As we expected, Figure 3a,c,d proved the presence of Ti, which in the case of the TPU–TiO_2_ NW composites confirms the embedding TiO_2_ NWs in the TPU matrix, inducing a fibrous structure in the free-standing films.

### 3.2. Structural Properties of TiO_2_ NWs and the TPU–TiO_2_ Composites

**Figure 4:**
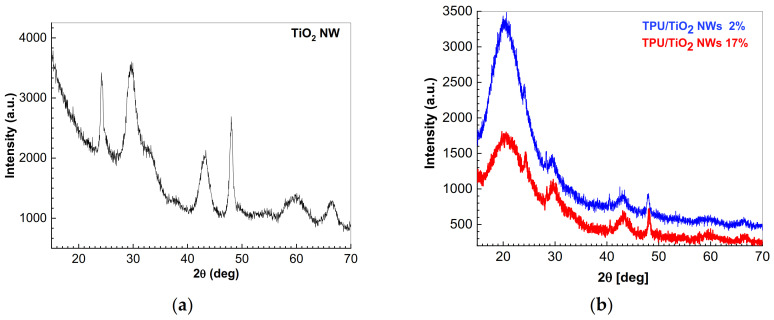
The XRD patterns of TiO_2_ NWs (**a**) and the TPU–TiO_2_ NW composites (**b**) with TiO_2_ NW concentrations equal to 2 wt.% (blue curve in Figure 4b) and 17 wt.% (red curve in Figure 4b).

Figure 4 shows the XRD patterns of TPU and the TPU–TiO_2_ NW composites, which have TiO_2_ NW concentrations equal to 2 wt.% and 17 wt.%, respectively. Figure 4a highlights the XRD patterns of TiO_2_ NWs peak at 24.2°, 29.7°, 43.4°, 48°, 59.9°, and 66.5° in two theta. According to the standard International Centre for Diffraction Data (ICDD) database, the first two peaks situated at 24.2° and 29.7° belong to the crystalline (110) and (310) planes of the monoclinic (M) H_2_Ti_3_O_7_ [44], PDF no.00-041-0192, while the peaks localized at 43.4° and 48° were assigned to the (200) plane in Ti_0.91_O, which has a cubic (C) crystalline structure [PDF no.04-004-2981], and the (200) plane in TiO_2_ of the type tetragonal anatase (A) [45,46], PDF no. 00-021-1272, respectively. The peaks around 60.2° and 66.3° in 2θ belong to the (501) and (404) planes, respectively, of a Ti_2_O_3_ phase with an orthorhombic (OR) crystalline structure (PDF04-018-9746).

Compared with Figure 4a, in the case of the TPU–TiO_2_ NW composites with TiO_2_ NW concentrations equal to 2 wt.% and 17 wt.%, one observes that: (i) the gradual increase in the added TiO_2_ NWs is clearly visible at the NWs’ specific 2θ angles; (ii) the peak in the range 15–25° (Figure 4b) belongs to TPU [43]; (iii) a shift in the peaks from 43.4° and 48° (Figure 4a) to 43.5° and 48.3° (blue curve in Figure 4b) or 43.6° and 48.1° (red curve in Figure 4b) occurs; and (iv) there is a change in the ratio between the intensities of the peaks at 43.4–43.6° and 48–48.3° from 0.78 (Figure 4a) to close to 1, i.e., 1.03 (blue curve in Figure 4b) and 0.96 (red curve in Figure 4b). This decrease in the intensity of the peak at 48–48.3° can only be explained if an interaction of TiO_2_ NWs with PTU occurs, when we estimate that the formation of suboxides takes place. To prove this claim, correlated Raman scattering and FTIR spectroscopy studies are presented below.

### 3.3. Vibrational Properties of TPU and the TPU–TiO_2_ NW Composites

**Figure 5:**
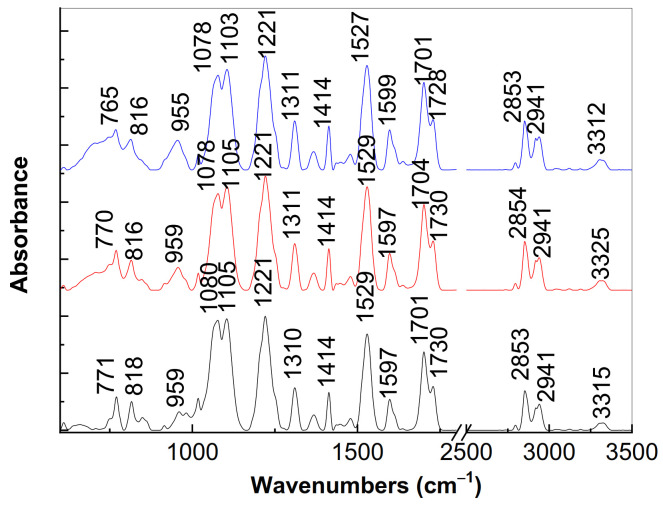
IR spectra of TPU (**a,** black curve) and TPU–TiO_2_ NW composites, which have TiO_2_ NW concentrations equal to 2 wt.% (**b**, red curve) and 17 wt.% (**c**, blue curve).

**Figure 6:**
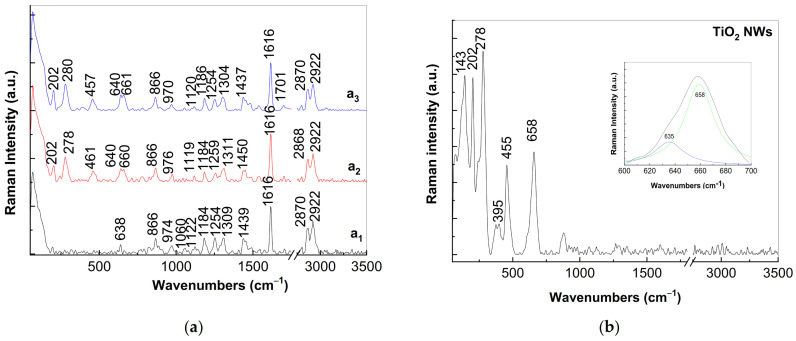
Raman spectra of: (**a**) TPU (**a_1_**); TPU–TiO_2_ NW composites have TiO_2_ NW concentrations equal to 2 wt.% (**a_2_**) and 17 wt.% (**a_3_**); and (**b**) TiO_2_ NWs.

Figure 5 and Figure 6 show the IR and Raman spectra of the TPU–TiO_2_ NW composites with TiO_2_ NW concentrations equal to 2 wt.% and 17 wt.%. The main IR bands of TPU peaked at 771, 818, 959, 1080–1105, 1221, 1310, 1414, 1529, 1597, 1701, 1730, 2853–2941, and 3315 cm^−1^ (Figure 5a). They are assigned to the vibrational modes of the N-H bond, C-H bond, higher-order aggregates, stretching C(O)–OC, CO stretching in the ether group, C-N bond, C-H bond, urethane group, C-C + C=C bonds in the benzene ring, hydrogen-linked urethane carbonyl group (C=O), the free carbonyl group, the antisymmetric and symmetrical vibrational modes of the CH bonds, and a partial inter- and intramolecular hydrogen linkage of the NH groups of the adjacent urethane segments [47,48,49,50,51,52,53], respectively.

Figure 5 shows that as the TiO_2_ NW concentration in the mass of the TPU–TiO_2_ NW composites increases: (i) there is a shift of the IR band from 771 cm^−1^ (Figure 5a) to 765 cm^−1^ (Figure 5c); (ii) a shift of the IR band from 959 cm^−1^ (Figure 5a) to 955 cm^−1^ (Figure 5b,c), the variation accompanied by a decrease in the ratio between the absorbance of the IR bands from 771–765 and 955–959 cm^−1^ from 1.82 (Figure 5a) to 1.75 (Figure 5b) and 1.32 (Figure 5c); (iii) a shift in the IR band from 3315 cm^−1^ (Figure 5a) to 3304 cm^−1^ (Figure 5c); and (iv) a variation in the ratio between the absorbance of the IR bands peaked at 765–771 cm^−1^ and 3304–3315 cm^−1^ from 4.68 (Figure 5a) to 4.21 (Figure 5b) and 3.83 (Figure 5c). These variations indicate a decrease in the higher-order aggregates of TPU simultaneous with the increase in the hydrogen bonds established between the amide groups of TPU and oxygen atoms of TiO_2_ NWs.

Additional information concerning the chemical structure of the TPU–TiO_2_ NW composites is shown in Figure 6. The main Raman lines of TPU are situated at 638, 866, 974, 1060, 1122, 1184, 1254, 1309, 1439, 1616, and 2870–2922 cm^−1^ (Figure 6a_1_), and they are assigned to the following vibrational modes: O-C=O in-plane deformation, out-of-plane benzene-ring deformation, out-of-plane C-H wagging, C-C skeletal stretching in alkane group, C-O-C, urethane amide, urethane amide III, deformation of the C-H bond in urethane amide III, symmetric stretching of N=C=O + deformation of CH_2_ group, stretching of the bonds C-C + C=C in aryl ring, and stretching of CH bond in aromatic structure [54,55], respectively.

**Scheme 1:**
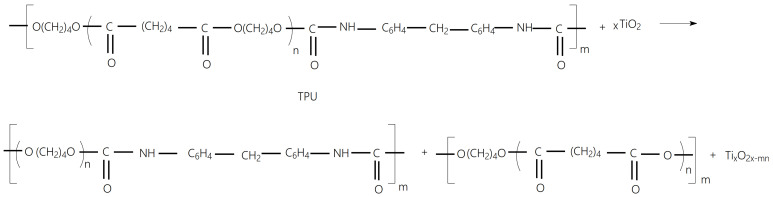
Exchange reaction of TPU in presence of TiO_2_ NWs.

**Scheme 2:**
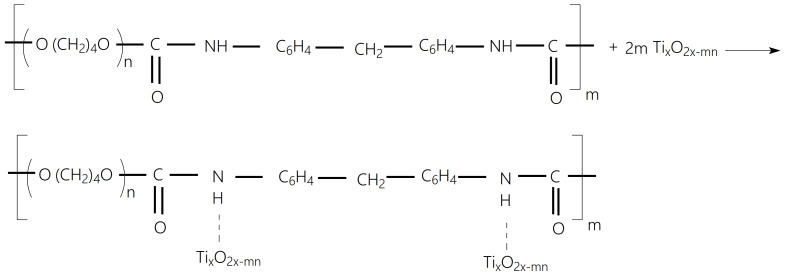
Physical adsorption of polymer with repeating units containing amide groups onto Ti_x_O_2x-mn_ surface.

Figure 6b shows that the main Raman lines of TiO_2_ NWs are localized at 143, 202, 278, 395, 455, and 658 cm^−1^, and they are assigned to the vibrational modes E_g_ in TiO_2_ A [56,57], stretching O-Ti-O in Ti_2_O_3_ [58], O-Ti-O in Ti_n_O_2n-1_ [58], B_1g_ in TiO_2_ A [56], A_1g_ in H_2_Ti_3_O_7_ [44], and E_g_ in H_2_Ti_3_O_7_, respectively [44]. Increasing the concentration of TiO_2_ NWs in the mass of the TPU–TiO_2_ NW composites induces an increase in the intensity of the Raman lines peaking at 278–280 cm^−1^, an upshift of the Raman line from 455 cm^−1^ (Figure 6b) to 457–461 cm^−1^ (Figure 6a_2_,a_3_), and a change in the profile of the Raman line at 658 cm^−1^ (Figure 6b). The insert in Figure 6b shows that the Raman line at 658 cm^−1^ displays an asymmetric profile to small wavenumbers as a consequence of the presence of a Raman line peaked at 635 cm^−1^, belonging to the vibrational mode E_g_ of H_2_Ti_3_O_7_ [56]. Regarding the Raman spectra of TiO_2_ NWs, the ratio between the intensity of the Raman lines peaked at 658 cm^−1^ and 635 cm^−1^, which were assigned to the vibrational modes E_g_ in TiO_2_ A and E_g_ in H_2_Ti_3_O_7_ (I_TiO2-A_/I_H2Ti3O7_), respectively, is equal to 3.45. Regarding the Raman spectra of the TPU–TiO_2_ NW composites with the TiO_2_ NW concentrations of 2 wt.% and 17 wt.%, the I_TiO2-A_/I_H2Ti3O7_ ratio is equal to 0.94 (Figure 6a_2_) and 0.96 (Figure 6a_3_), respectively. The decrease in the value of the I_TiO2-A_/I_H2Ti3O7_ ratio in the case of the TPU–TiO_2_ NW composites indicates a diminution of TiO_2_ A in the TiO_2_ NWs mass. This fact can be explained by taking into account the exchange reaction of TPU according to Scheme 1, which is followed by the formation of new hydrogen bonds between the NH bonds of the amide groups and the oxygen atoms of Ti_x_O_2x-mn_ (Scheme 2).

The reaction products of Scheme 1 can be described as follows: (a) the first corresponds to a macromolecular compound with amide groups in the repeating units; (b) the second corresponds to a macromolecular compound characterized by the repeating units having acetate groups; and (c) the third corresponds to suboxide Ti_x_O_2x-mn_.

Similar to Scheme 2, new hydrogen bonds emerge between the -NH- bond of the TPU amide group and the oxygen atoms of Ti_x_O_2x-mn_. Such hydrogen bonds can be invoked to occur between the -NH- bonds of the TPU amide groups and the oxygen atoms of Ti_2_O_3_ and Ti_0.91_O.

Summarizing these results, the exchange reaction presented in Scheme 1 is confirmed by the change in the ratio between the intensities of the peaks at 43.4–43.6° and 48–48.3° from 0.78 (Figure 4a) to close to 1, i.e., 1.03 (blue curve in Figure 4b) and 0.96 (red curve in Figure 4b), which indicates the emergence of suboxide Ti_x_O_2x-mn_, as well as the decrease in the ratio between the intensity of the Raman lines peaked at 658 cm^−1^ and 635 cm^−1^, which is assigned to the vibrational modes E_g_ in TiO_2_ A and E_g_ of H_2_Ti_3_O_7_, from 3.45 to 0.94–0.95 in the case of TPU and the TPU–TiO_2_ NW composites (Figure 6). This indicates that there is a decrease in the higher-order aggregates as a consequence of the emergence of new hydrogen bonds between the -NH-, which belongs to the TPU amide groups, and the oxygen atoms of Ti_x_O_2x-mn_, Ti_2_O_3_, and Ti_0.91_O.

### 3.4. Photoluminescence of TPU and the TPU–TiO_2_ NWs Composites

**Figure 7:**
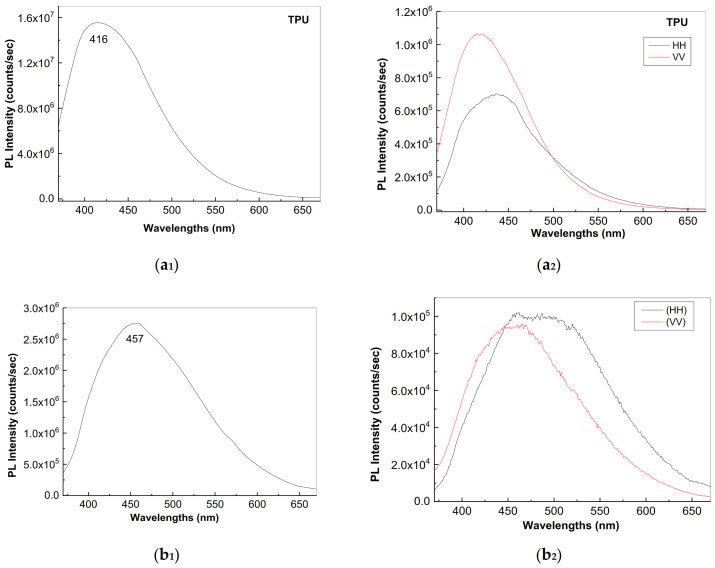

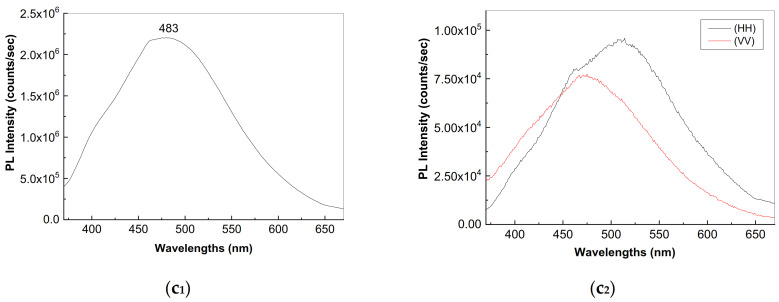
PL spectra of TPU (**a_1_**), PTU–TiO_2_ NWs 2% (**b_1_**), and PTU–TiO_2_ NWs 17% (**c_1_**). Anisotropic PL of TPU (**a_2_**), PTU–TiO_2_ NWs 2% (**b_2_**), and PTU–TiO_2_ NWs 17% (**c_2_**). In (**a_2_**,**b_2_**,**c_2_**), blue and red curves correspond to PL spectra recorded when measurement geometry for emission and excitation polarizers are both in horizontal (HH) and vertical (VV) position.

Figure 7 shows the PL spectra of TPU and the TPU–TiO_2_ NW composites have a TiO_2_ NW concentration equal to 2 wt.% and 17 wt.%. The increase in the TiO_2_ NW concentration in the TPU–TiO_2_ NW composites mass from 0 wt.% to 17 wt.% induces a shift in the maximum level of the emission band from 416 nm (Figure 7a_1_) to 457 nm (Figure 7b_1_) and 480 nm (Figure 7c_1_), as well as a decrease in the intensity of the PL band from 1.55 × 10^7^ counts/sec (Figure 7a_1_) to 2.76 × 10^6^ counts/sec (Figure 7b_1_) and 2.2 × 10^6^ counts/sec (Figure 7c_1_). This decrease in the PL band indicates that TiO_2_ NWs are PTU PL quenching agents. According to G. Strat et al., the redshift of the TPU’s PL band results from the luminescence centers belonging the small-order aggregates [59], which in our case appear as a consequence of the reactions shown in Scheme 1 and Scheme 2.

Using the mathematic protocol reported in [60], the calculated values of the anisotropy (r) and binding angle (θ_PL_) for TPU are 0.3712 and 12.6°, respectively, while the PTU–TiO_2_ NW composite has a TiO_2_ NW concentration equal to 2 wt.%, which is 0.2315 and 32°, and the PTU–TiO_2_ NW composite has a TiO_2_ NW concentration of 17 wt.%, which is 0.0905 and 45.9°. These values indicate that increasing the TiO_2_ NW concentration in the PTU–TiO_2_ NW composite mass results in an increase in θ_PL_ of TPU onto the TiO_2_ NW surface. As shown above, the θ_PL_ values are different from 0°, which suggests that the TPU’s excitation and emission transition dipoles are not parallel with the TiO_2_ NW plane. The orientation of TPU onto the TiO_2_ NW surface must consider the hydrogen bonds established between TiO_2_ NWs and TPU as well as the products of TPU’s exchange reaction with TiO_2_ NWs.

## 4. Conclusions

In this work, we reported new results concerning the optical and structural properties of TPU–TiO_2_ NW composites as free-standing films. Our results highlight the following conclusions: (i) using X-ray diffraction, we demonstrated that TiO_2_ NWs contain TiO_2_ anatase (A), Ti_0.91_O and Ti_2_O_3_ have cubic (C)- and orthorhombic (OR)-type crystalline structures, respectively, while H_2_Ti_3_O_7_ has a monoclinic (M)-type structure; (ii) in the case of TPU–TiO_2_ NW composites, with TiO_2_ NW concentration 2 wt.% and 17 wt.%, the increase in the intensity of the diffraction peak localized at 43.2° indicated the formation of titanium suboxides; (iii) according to studies using FTIR spectroscopy, the interaction of TPU with TiO_2_ NWs involved a decrease in the higher-order aggregates of TPU simultaneous with an increase in the hydrogen bonds established between the TPU amide groups and oxygen atoms of TiO_2_ NWs, facts that were highlighted by the variation of the ratio between the absorbance of the IR bands peaking at 765–771 cm^−1^ and 3304–3315 cm^−1^ from 4.68 to 3.83 when the concentration of TiO_2_ NWs in the composite mass was 0 wt.% and 17 wt.%; (iv) according to Raman spectroscopy, the decrease in the ratio between the intensity of the Raman lines peaked at 658 cm^−1^ and 635 cm^−1^, which were assigned to the vibrational modes E_g_ in TiO_2_ A and E_g_ in H_2_Ti_3_O_7_ (I_TiO2-A_/I_H2Ti3O7_), respectively, from 3.45 in TiO_2_ NWs to 0.94–0.96 in the TPU–TiO_2_ NW composites, indicating that the adsorption of TPU onto TiO_2_ NWs involves an exchange reaction of TPU in the presence of TiO_2_ NWs, which is followed by the formation of new hydrogen bonds between the -NH- of the amide group and the oxygen atoms of Ti_x_O_2x-mn_, Ti_2_O_3_, and Ti_0.91_O; (v) we demonstrated that the TiO_2_ NWs are TPU PL quenching agents, which was evidenced by the decrease in the intensity of the emission band of the TPU–TiO_2_ NW composite, which was localized in the spectral range 380–650 nm as increasing TiO_2_ NW concentration; and (vi) anisotropic photoluminescence studies indicated a preferential orientation of TPU onto the TiO_2_ NW surface, such as when increasing the TiO_2_ NW concentration in the PTU–TiO_2_ NW composite mass from 0 wt.% to 2 wt.% and 17 wt.%, which induced the increase in the polymer binding angle onto the TiO_2_ NW surface (θ_PL_) from 12.6° to 32° and 45.9°.

## Data Availability

The data reported in this manuscript are available upon request from the corresponding author.
